# Four‐dimensional computed tomography‐based ventilation imaging in intensity‐modulated radiation therapy treatment planning for pulmonary functional avoidance

**DOI:** 10.1002/acm2.13920

**Published:** 2023-02-02

**Authors:** Gazi Md Daud Iqbal, Hao Zhang, Wareen D'Souza, Lidan Ha, Jay M. Rosenberger

**Affiliations:** ^1^ College of Business Coppin State University Baltimore Maryland USA; ^2^ University of Maryland Medical Systems Linthicum Maryland USA; ^3^ Industrial, Manufacturing, & Systems Engineering University of Texas at Arlington Arlington Texas USA

**Keywords:** voxel based treatment, column generation algorithm, lung cancer optimization

## Abstract

**Purpose:**

To incorporate four‐dimensional computed tomography (4DCT)‐based ventilation imaging into intensity‐modulated radiation therapy (IMRT) treatment planning for pulmonary functional avoidance.

**Methods and Materials:**

Nineteen locally advanced lung cancer patients are retrospectively studied. 4DCT images are employed to create ventilation maps for each patient via a density‐change‐based algorithm with mass correction. The regional ventilation is directly incorporated into the mathematical formulation of a direct aperture optimization model in IMRT treatment planning to achieve functional avoidance and a voxel‐based treatment plan. The proposed functional avoidance planning and voxel‐based planning are compared to the conventional treatment planning approach purely based on the anatomy of patients. Paired sample *t*‐tests are conducted to see whether dosimetric differences among the three approaches are significant.

**Results:**

Similar planning target volume (PTV) coverage is achieved by anatomical, functional avoidance, and voxel‐based approaches. The voxel‐based treatment planning performs better than both functional avoidance and anatomical planning to the lung. For a total lung, the average volume reductions in a functional avoidance plan from an anatomical plan, a voxel‐based plan from an anatomical plan, and a voxel‐based plan from a functional avoidance plan are 7.0%, 16.8%, and 10.6%, respectively for V_40_; and 0.4%, 6.4%, and 6.0%, respectively for mean Lung Dose (MLD). For a functional lung, the reductions are 8.8%, 17.2%, and 9.2%, respectively, for fV_40_; and 1.1%, 6.2%, and 5.2%, respectively, for functional mean lung dose (fMLD). These reductions are obtained without significantly increasing doses to other organs‐at‐risk. All the pairwise treatment planning comparisons for both total lung and functional lung are statistically significant (*p*‐value <α=0.05) except for the functional avoidance plan with the anatomical plan pair in which the *p*‐value >α=0.05. From these results, we can conclude that voxel‐based treatment planning outperforms both anatomical and functional‐avoidance planning.

**Conclusions:**

We propose a treatment planning framework that directly utilizes functional images and compares voxel‐based treatment planning with functional avoidance and anatomical treatment planning.

## 1 INTRODUCTION

Pulmonary ventilation imaging provides useful information on regional lung function. An emerging technique to compute lung ventilation map has been proposed^[^ ^1^, ^2^ ^]^ that uses four‐dimensional computed tomography (4DCT) image datasets. Because 4DCT images are routinely acquired for lung cancer patients in current clinical practice, ventilation computation involves mere imaging processing. Compared to other imaging modalities, such as single photon emission computed tomography (SPECT)^[^ ^3^ ^]^ and magnetic resonance (MR)^[^ ^4^ ^]^, 4DCT ventilation imaging has a higher resolution, lower cost, shorter scan time, and greater availability.^[^ ^5^ ^]^

Research on 4DCT‐based ventilation imaging has focused on its methodology,^[^ ^6^ ^]^ validation and clinical usage. Several recent validation studies^[^ ^5^, ^7^, ^8^ ^]^ have compared 4DCT‐based ventilation with both regional nuclear medicine ventilation‐perfusion imaging and the global pulmonary function test, and generally showed good agreement with the different techniques in assessing lung function. With respect to clinical usage, Vinogradskiy et al.^[^ ^9^ ^]^ uses 4DCT‐based ventilation maps to evaluate lung function changes throughout the course of radiotherapy treatment, and Vinogradskiy et al.^[^ ^10^ ^]^ incorporates 4DCT ventilation‐based function to better correlate lung dose with radiation pneumonitis. Another clinical use of 4DCT‐based ventilation map was to create functional avoidance plans.^[^ ^11^, ^12^ ^]^ These studies generally involved generating a new contour corresponding to a highly functional lung region based on the ventilation map and imposing new dose‐volume constraints on the contour so that desired doses would be delivered to the region. Although this approach did take into account heterogeneous lung function, it took a binary view of the entire function spectrum, essentially assuming function uniformity in the high‐functioning lung as well as the low‐functioning lung.

Our proposed methodology in this study instead takes full advantage of the entire function spectrum provided by the ventilation map and directly incorporates lung ventilation at every voxel into a mathematical model for inverse planning. We use regional ventilation to modulate the penalty scalars associated with lung overdose on a voxel‐by‐voxel basis so that the voxels with high function are given relatively larger penalties than those with low function. This could thus potentially steer dose away from well‐ventilated lung regions.

We remark that the proposed methodology is similar to that of St‐Hilaire et al.^[^ ^13^ ^]^ But instead of using SPECT perfusion information in an aperture‐based IMRT model, we consider 4DCT‐based ventilation in a beamlet‐based model.

## 2 METHODS

### 2.1 Patient CT and contours

Nineteen locally advanced lung cancer patients treated at University of Maryland School of Medicine are retrospectively studied. Each of these patients has a free‐breathing CT and 4DCT scan prior to treatment. The gross tumor volume (GTV), clinical target volume (CTV), internal target volume (ITV), planning target volume (PTV), and organs‐at‐risk (OARs), including heart, esophagus, spinal cord, and total lung (minus GTV) are contoured on the planning CT image. The GTV to CTV margin is 6 mm. The ITV is obtained by adding a margin of 6 mm isotropically to the CTV, and the PTV is formed by expanding the ITV isotropically by a margin of 5 mm.

### 2.2 4DCT‐based ventilation imaging

Using the 4DCT image data set for each patient, a ventilation map is calculated via a density‐change‐based algorithm with mass correction.^[^ ^2^ ^]^ Lungs are segmented on the CT images at the inhale and exhale phases of the respiratory cycle. The images at these two phases are matched to each other using a deformable image registration technique. Let $V_{\mathrm{IN}}$ and $V_{\mathrm{EX}}$ be the inhale and exhale volumes for the voxel, and $\rho_{\mathrm{IN}}$ and $\rho_{\mathrm{EX}}$ are the voxel densities at the inhale and exhale phases. Let parameter *k* be the ratio of the entire lung mass between the inhale and exhale phases. It is introduced in Equation (1) to account for the tissue‐based mass changes that occur over the respiratory cycle. The local ventilation for a given voxel is estimated by

(1) $$\begin{array}{c} \frac{V_{\mathrm{IN}}-V_{\mathrm{EX}}}{V_{\mathrm{EX}}}=\frac{k\rho_{\mathrm{EX}}-\rho_{\mathrm{IN}}}{\rho_{\mathrm{IN}}} \end{array}$$

A lung can be treated as a composite of mainly air and tissue. Let $V_{\mathrm{VOX}},V_{\mathrm{AIR}}$, and $V_{\mathrm{TISSUE}}$ be the volumes for the voxel and its air and tissue components, respectively. The density of a voxel is approximated by

(2) $$\begin{array}{c} \rho_{\mathrm{VOX}}=\frac{\rho_{\mathrm{AIR}}V_{\mathrm{AIR}}+\rho_{\mathrm{TISSUE}}V_{\mathrm{TISSUE}}}{V_{\mathrm{VOX}}} \end{array}$$

where $\rho_{\mathrm{AIR}}\approx 0.001g/cm^{3}$ and $\rho_{\mathrm{TISSUE}}\approx 1.1g/cm^{3}$.

### 2.3 Monte‐Carlo dose calculation

An in‐house Monte Carlo Kernel‐based Superposition (MCKS) dose calculation engine is applied to compute beamlet dose distributions for each beam orientation. The dose deposited to every voxel in each structure (PTV or OAR) by each beamlet (namely the dose deposition matrix) is obtained as input to the mathematical model for inverse planning. The voxel size is 4 mm × 4 mm × 2.5 mm. The same beam orientations that are used clinically to treat the patients are used in this study, which ranged from 4 to 10. The beamlet size is 1 cm by 1 cm. Depending on the anatomy of the patient, a field size of 12 cm × 12 cm, 16 cm × 16 cm or 20 cm × 20 cm is calculated to ensure adequate coverage of the tumor for each patient. Thus the total number of beamlets is between 576 and 2560.

### 2.4 Mathematical model

Let *S* be the set of all structures in the treatment volume. Each treatment volume consists of target structures (PTV), organs‐at‐risk (OARs), critical structures, and normal tissues. Esophagus, heart, and lung are examples of OARs, while the brain and spinal cord are in the critical structures category. Let T⊆S be the set of target structures, O⊆S be the set of OARs, and C⊆S be the set of critical structures. Let $V_{s}$ be the set of voxels for each s∈S. Let *B* be the set of beam angles. Each beam consists of *m* rows and *n* columns and forms a rectangular grid of beamlets. A two‐dimensional matrix, which is called intensity matrix, is often used to specify the position and intensity of all beamlets. Each element of this intensity matrix is called a bixel. Let *N* be the set of all bixels where N=B×{1,..,m}×{1,..,n}. let $K_{l}$ be the set of allowable apertures for each beam l∈B, let $K\equiv \cup_{l\in B}K_{l}$ be the set of all apertures, and let $A_{k}\subset N$ be the set of beamlets that are exposed in aperture k∈K. Let parameter $D_{ils}$ be the dose deposition matrix for all $i\in A_{k},l\in V_{s},s\in S$. Let decision variables $a_{k}$ and $z_{ls}$ be the weight of aperture k∈K, and dose received by each voxel $l\in V_{s}$ and s∈S, respectively. $z_{ls}$ is defined as follows :

(3) $$\begin{array}{ccc} z_{ls}=\sum_{k\in K}\sum_{i\in A_{k}}a_{k}D_{ils} & & \forall \;l\in V_{s},s\in S \end{array}$$

Similar to this research, the optimization model in Romeijn et al.^[^ ^14^ ^]^ describes a column generation approach to radiation therapy treatment planning using aperture modulation. They used head‐and‐neck cancer patients data to study the quality of the treatment plans generated using this optimization approach. However, in this research we use lung cancer patients data. The weighted optimization model (hereafter **restricted problem**) is shown below:

(4) $$\begin{array}{ccc} & & \min\;w_{1}\left(\sum_{s\in T}\max_{l\in V_{s}}\left|z_{ls}-pd_{s}\right|\right)+w_{2}\left(\sum_{s\in O}\frac{1}{|V_{s}|}\sum_{l\in Vs}z_{ls}\right)\\ & & \;+\;w_{3}\left(\sum_{s\in C}\max_{l\in V_{s}}z_{ls}\right) \end{array}$$

(5) $$\begin{array}{ccc} \text{subject to:}\;\;(3) & & \\ \zeta_{s}-\frac{1}{\left(1-\alpha_{s}|V_{s}|\right)}\sum_{l\in V_{s}}\max\left(\zeta_{s}-z_{ls},0\right)\geq pd_{s} & & \forall s\in T \end{array}$$

(6) $$\begin{array}{ccc} a_{k}\geq 0, & & \forall k\in K \end{array}$$

Objective (4) is the weighted sum of target structures, OARs, and critical organs. For target structures, our goal is to minimize the maximum variation from the prescribed dose $pd_{s}$. We want to minimize the mean dose for OARs. For critical organs, we wish to minimize the max dose. The rest of the volume of the treatment region is the normal tissue. We wish to minimize the maximum dose for normal tissue. Constraint set (5) guarantees that $\alpha_{s}$ percentage of target structure receives the prescribed dose $pd_{s}$, and $\zeta_{s}$ is a free variable for all s∈T.^[^ ^14^ ^]^ Constraint set (6) ensures the non‐negativity of aperture weights.

The number of apertures produced in the restricted problem is much too large. However, it is expected that only few apertures will actually be used.^[^ ^14^ ^]^ In this research, we start solving restricted problem by choosing a limited set of apertures. Then to check the optimality of the restricted problem, a subproblem called the pricing problem is solved to try to identify the apertures to enter into the restricted problem.^[^ ^15^ ^]^

Let binary variable $x_{i}$ represents an aperture such that

$$x_{i}=\left\{\begin{array}{cc} 1, & \text{if beamlet}\;i\in N\;\text{is exposed in the aperture,}\\ 0, & \text{otherwise.} \end{array}\right.$$

Let *X* be the set of all binary vectors *x* that represents an allowable aperture. Then the pricing problem is defined as follows:

(7) $$\begin{array}{c} \min_{x\in X}\sum_{i\in N}\left(\sum_{s\in S}\sum_{l\in V_{s}}D_{ils}\pi_{ls}\right)x_{i} \end{array}$$

If the value of the above pricing problem (7) is nonnegative, then the current solution is optimal. Otherwise, we include the generated new aperture to the restricted problem. We then re‐optimize the new restricted problem and repeat it until there are no such apertures left that can improve the solution of the restricted problem.

### 2.5 Plan generation

The clinical dose‐volume goals for the target volume and OARs are listed in Table 1. These goals are applied in anatomical (hereafter **Anatom**), functional avoidance (hereafter **FuncAvoid**), and voxel‐based (hereafter **Voxel**) plans as described in the previous section. The target prescription dose D_pres is 70 Gy. All the treatment plans were normalized so that 95% of the PTV received the prescription doses. 100% of the ITV and GTV received the prescription dose.

**TABLE 1:** Clinical dose‐volume goals for target volume and organs‐at‐risk

Structure	Specification	Dose (Gy)	Volume(%)
PTV	Minimum DVH	D_pres	95
Lung	Maximum DVH	[15,18] Gy	
Spinal cord	Maximum dose	[40,45] Gy	
Heart	Maximum DVH	[10,15] Gy	
Esophagus	Maximum DVH	[30,34] Gy	
High functional lung	Maximum DVH	[15,20] Gy	
Mid functional lung	Maximum DVH	[30,34] Gy	
Low functional lung	Maximum DVH	[40,50] Gy	
Normal tissue		[0,1.15 PD_s]	

All procedures are coded in the C++ programming language, and IBM ILOG CPLEX Callable library is used to solve the optimization model described above. By successively adjusting the penalty weights of all the structures associated with individual dose‐volume goals in the objective function, the Anatom, FuncAvoid, and Voxel treatment plans are alternately solved to

Abbreviations: DVH, dose‐volume histogram; PTV, planning target volume.

improve target coverage and OAR sparing. The adjustments for each model are also guided by and made to emulate the best plan results from the other model. The process terminates when successive improvement of plan quality for either model is sufficiently small. This approach of plan generation is similar to what was used by Rao et al.^[^ ^16^ ^]^ in an effort to make the ensuing plan comparison as fair as possible. The final dose distributions for both plans are normalized so that 95% of the PTV receive the prescription dose.

An Anatom plan is achieved by equal weighting of all the structures in the objective function. Three structures are included in FuncAvoid plan, which are high functional, medium functional, and low functional region. Functional voxels are normalized between 0 and 1. Low, medium, and high functional structures are in the region of 0∼33%, >33%∼66%, and >66%∼100%, respectively. Voxel plan is achieved by adjusting weights for lungs as a function of ventilation mapping. Weights are adjusted by trial and error methods for all the plans.

Fixed field IMRT technique was used with 6 MV Varian TrueBeam machine. This is a retrospective study. We used the exact same clinical beam setup following clinical protocols, and the exact same fields were used as the anatomic treatment plan in the clinic.

### 2.6 Statistical analysis

To compare the Anatom, FuncAvoid, and Voxel treatment plans, dose‐volume histograms (DVHs) for the PTV and OARs and lung dose‐function histograms (DFH)^[^ ^17^ ^]^ are computed. DVH and DFH parameters collected for comparison include lung V_20_, V_30_, V_40_, mean lung dose (MLD), and their functional counterparts, mean heart dose, mean esophagus dose, maximum dose delivered to the spinal cord, and PTV uniformity index. The functional mean lung dose (fMLD) was computed for each plan as

$$\begin{array}{c} \mathrm{fMLD}=\frac{\sum_{v\in L}f_{v}D_{v}}{\sum_{v\in L}f_{v}} \end{array}$$

where $f_{v}$ and $D_{v}$ denote the local ventilation and delivered dose at voxel *v*, respectively, and *L* is the set of voxels in the lung. The PTV uniformity index is defined as the ratio of the dose providing 95% coverage of the PTV to the dose providing 5% coverage of the PTV. It is calculated using following formula given in Prabhakar.^[^ ^18^ ^]^

$$\begin{array}{c} \mathrm{Uniformity}\;\mathrm{Index}=\frac{\sum_{i=\mathrm{PD}}^{\mathrm{Max}\;\mathrm{Dose}}{\mathrm{Dose}}_{i}\times \mathrm{Volume}\left({\mathrm{Dose}}_{i}\right)}{V_{\mathrm{presc}}\times \mathrm{PD}} \end{array}$$

where $\mathrm{Volume}({\mathrm{Dose}}_{i})$ is the volume receiving a given dose *i*, $V_{\mathrm{presc}}$ is the volume of the prescription isoline, and PD is the prescription dose. Note that due to plan normalization, the dose providing 95% coverage of the PTV is always equal to the prescription dose. A paired sampled *t*‐test is conducted to see whether there is significant difference at the collected DVH and DFH parameters between the two plans. A *p*‐value less than 0.05 (α‐value) is considered statistically significant.

## 3 RESULTS

**FIGURE 1:**
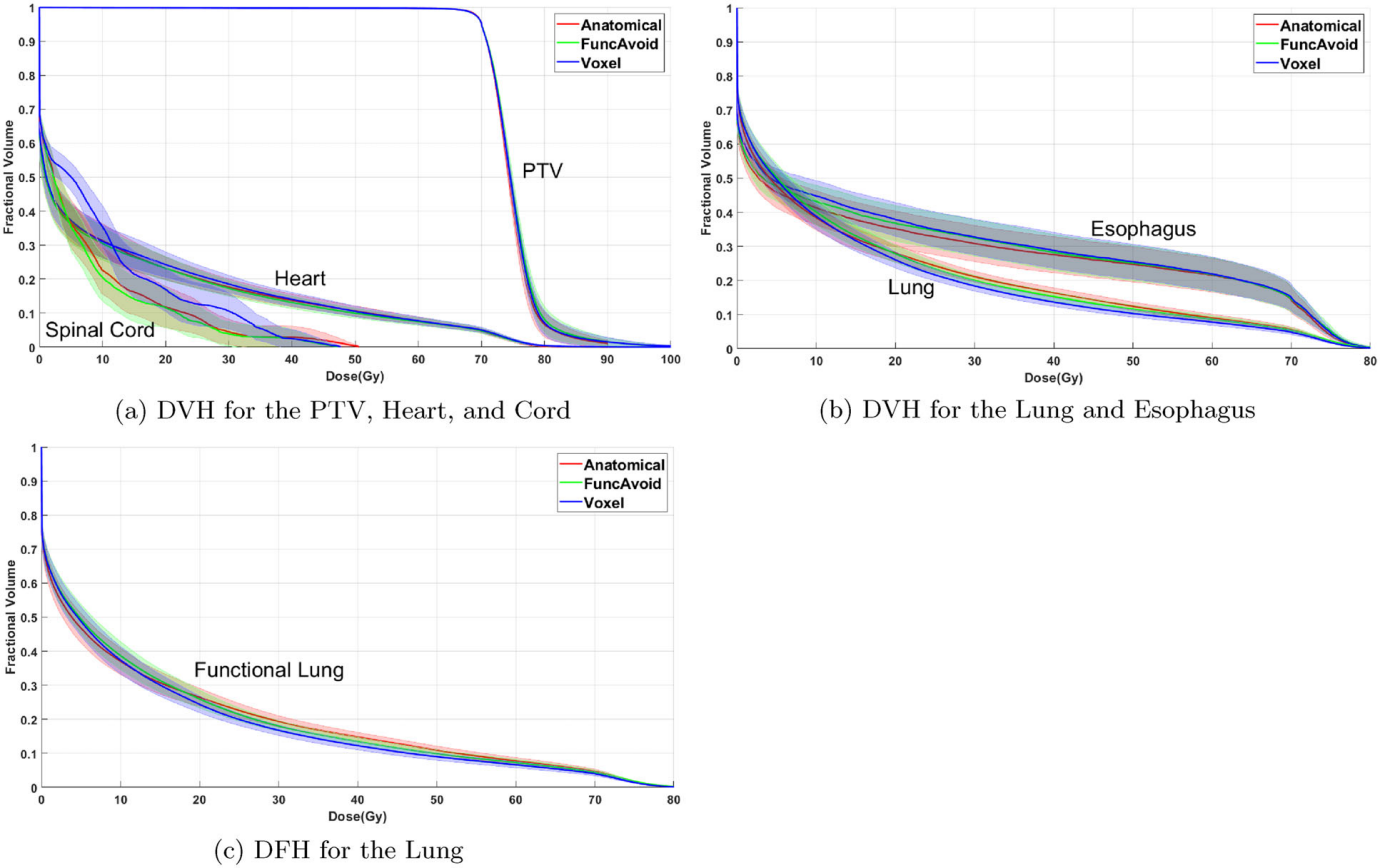
Dose‐volume histogram (DVH) for the planning target volume (PTV) and organs‐at‐risk, and dose‐function histogram (DFH) for the lung.

Figure 1 shows the dose‐volume histograms (DVHs) for the PTV, OARs, and lung dose‐function histogram (DFH) of the Anatom, FuncAvoid, and Voxel Intensity Modulated Radiation Therapy (IMRT) plans averaged over 19 patients. The Voxel plan evidently achieved more favorable DVH and DFH for the total and functional lung than the Anatom and FuncAvoid plan. With similar PTV coverage, the PTV uniformity index is 1.07 for anatomical, and 1.08 for both FuncAvoid and Voxel treatment plans.

**FIGURE 2:**
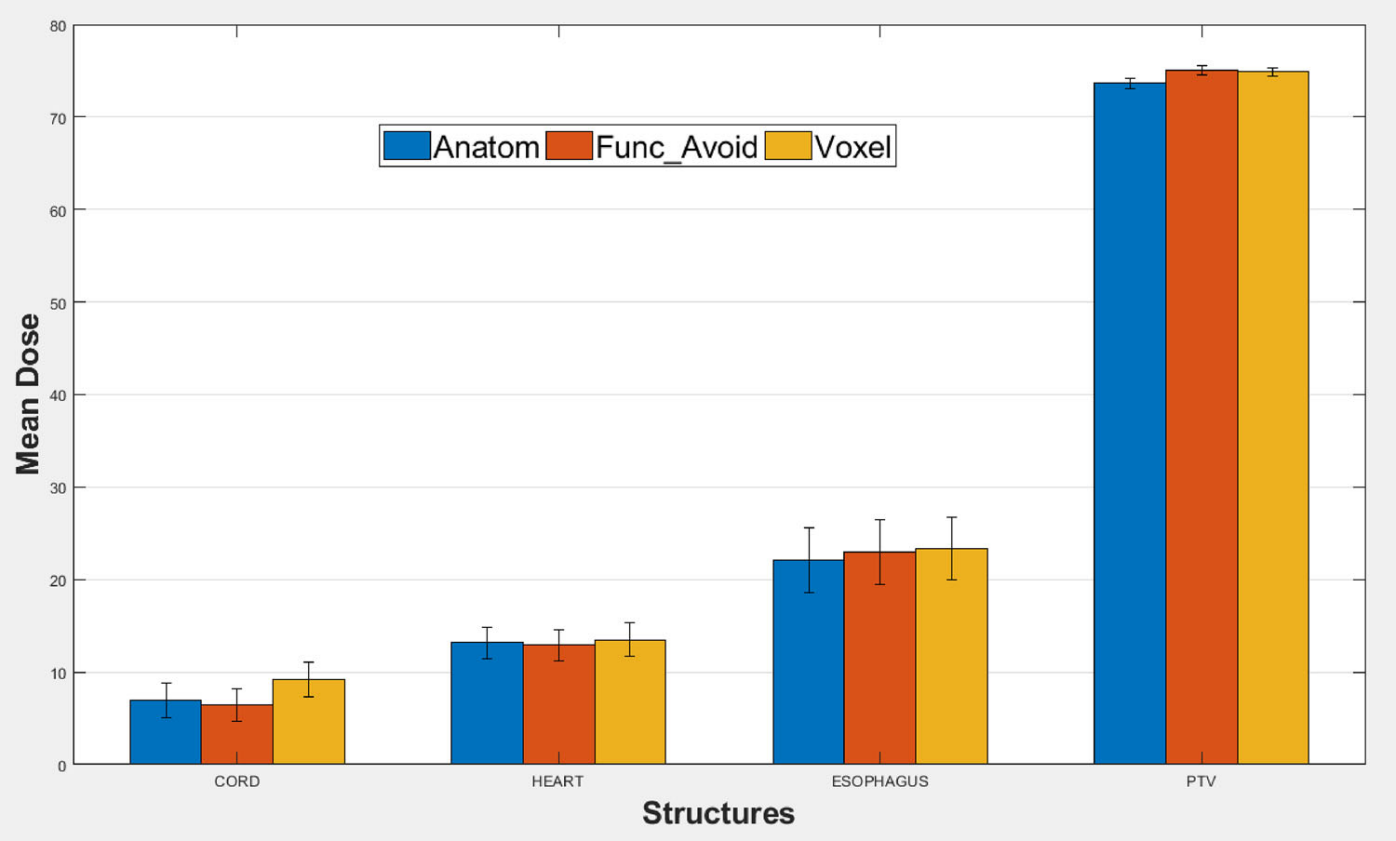
Comparison of mean doses of cord, heart, esophagus, and planning target volume (PTV) among the anatomical, functional‐avoidance, and voxel‐based planning.

Figure 2 shows the mean dose comparison of Anatom, FuncAvoid, and Voxel treatment planning for cord, heart, esophagus, and PTV. The dose delivered to the heart, esophagus, and PTV are similar in all three plans. Only cord gets slightly worsened in the Voxel treatment planning compared with the other two treatment plans. We also conduct paired sample t‐tests among all three treatment plans for PTV, heart, esophagus, and cord. f 2 shows the *t*‐test results. One thing to notice is that PTV has no mean differences among all the three plans. Other structures have a mix of significant and not‐significant results for pairwise comparisons.

**TABLE 2:** Paired‐sample *t*‐test for structures among three treatment plans

	p‐value		
Structures	Voxel ∼ Anatom	Voxel ∼ FuncAvoid	FuncAvoid ∼ Anatom
PTV, uniformity index	0.081	0.074	0.065
Heart, mean dose	0.063	<0.001	0.097
Esophagus, mean dose	<0.001	0.083	<0.001
Spinal cord, maximum dose	<0.001	<0.001	0.107

Abbreviation: ptv, planning target volume.

**FIGURE 3:**
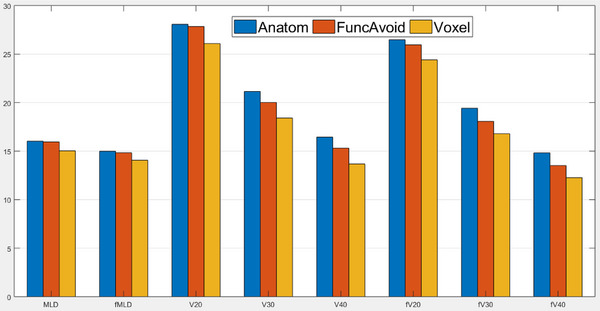
Comparison of mean lung dose (MLD), functional mean lung dose (fMLD), percentage of lung volume receiving 20, 30, and 40 Gy (V_20_, V_30_, and V_40_, respectively), percentage of functional lung volume receiving 20, 30, and 40 Gy (fV_20_, fV_30_, and fV_40_, respectively) among the anatomical, functional‐avoidance, and voxel‐based plans.

Lung DVH and DFH parameters in the Anatom, FuncAvoid, and Voxel plans for all patients are summarized in Figure 3. In this figure, the units in the Y‐axis are the actual mean dose for MLD and fMLD, while it is the volume receiving the dose for V_20_, V_30_, and V_40_. Voxel plan led to an overall significant decrease in lung dose compared to Anatom and FuncAvoid planning. For total lung, the average reductions in FuncAvoid plan from Anatom plan, Voxel plan from Anatom plan, and Voxel plan from FuncAvoid plan are 7.0%, 16.8%, and 10.6%, respectively, for V_40_; and 0.4%, 6.4%, and 6.0%, respectively, for mean lung dose (MLD). For functional lung, the reductions are 8.8%, 17.2%, and 9.2%, respectively for fV_40_; and 1.1%, 6.2%, and 5.2%, respectively for functional mean lung dose (fMLD). These average volume reductions are shown in Table 4. We conduct paired sampled t‐tests for V_20_, V_30_, V_40_, and MLD with their dose‐function counterparts, fV_20_, fV_30_, fV_40_, and fMLD for all three plans. Table 3 shows the *t*‐test results. The reductions in functional lung are statistically significant.

**TABLE 3:** Paired‐sample *t*‐test between different total lung volume and their dose‐function counterparts for all three plans

	*p*‐value		
Parameter pair	Anatom	FuncAvoid	Voxel
V_20_ ∼ fV_20_ (%)	0.001	<0.001	<0.001
V_30_ ∼ fV_30_ (%)	<0.001	<0.001	<0.001
V_40_ ∼ fV_40_ (%)	0.002	<0.001	0.001
MLD ∼ fMLD (%)	<0.001	<0.001	<0.001

**TABLE 4:** Paired‐sample *t*‐test and% of volume reduction for different volumes of total lung and functional lung among three treatment plans

	Anatom ∼ FuncAvoid		Anatom ∼ Voxel		FuncAvoid ∼ Voxel	
Parameter	*p*‐value	% of volume reduction	*p*‐value	% of volume reduction	*p*‐value	% of volume reduction
V_20_ (%)	0.481	0.8	<0.001	7.1	<0.001	6.3
V_30_ (%)	<0.001	5.3	<0.001	13.0	<0.001	8.0
V_40_ (%)	<0.001	7.0	<0.001	16.8	<0.001	10.6
MLD (%)	0.383	0.4	<0.001	6.4	<0.001	6.0
fV_20_ (%)	0.161	2.0	<0.001	7.8	<0.001	6.0
fV_30_ (%)	<0.001	7.0	<0.001	13.5	<0.001	7.0
fV_40_ (%)	<0.001	8.8	<0.001	17.2	<0.001	9.2
fMLD (%)	0.115	1.1	<0.001	6.2	<0.001	5.2

We also conduct paired‐sample *t*‐tests for V_20_, V_30_, V_40_, MLD, fV_20_, fV_30_, fV_40_, and fMLD to see whether there is any statistical significance among the three plans. Table 4 shows the *t*‐test results between FuncAvoid with Anatom, Voxel with Anatom, and Voxel with FuncAvoid plans. Almost all the pairs are significantly different except V_20_, MLD, and fMLD for the funcAvoid and Anatom plan pair.

## 4 DISCUSSION

This study demonstrates that the incorporation of 4DCT‐based ventilation imaging in IMRT treatment planning led to significant reductions in lung dose without significantly impacting doses to other organs‐at‐risk (OARs) or planning target volume (PTV) coverage.

A number of studies^[^ ^11^, ^12^, ^13^, ^19^, ^20^, ^21^, ^22^, ^23^ ^]^ investigate the utilization of functional imaging in radiotherapy treatment planning (RTP). These studies generally involve different treatment techniques (e.g., 3D conformal radiation therapy and IMRT) and/or functional imaging modalities (e.g., single photon emission computed tomography (SPECT) perfusion imaging, magnetic resonance (MR) and 4DCT‐based ventilation imaging). In addition, depending on how the regional function information is utilized, the optimization methods are rather different. For example, Seppenwoolde et al. ^[^ ^19^ ^]^ optimize the treatment plan by minimizing mean perfusion‐weighted lung dose derived from the perfusion‐based dose‐function histogram, while Christian et al.^[^ ^20^ ^]^ consider the planning objective to minimize the dose to a volume of functional lung contoured on the perfusion map.

In spite of these diversities, all the studies demonstrated that the incorporation of functional imaging into the RTP could improve functional sparing of the lung, at least for a particular subset of patients (e.g., patients with large perfusion deficit). The improvement was generally reflected by significant reductions in some dose‐function parameters on the total lung^[^ ^13^, ^19^ ^]^ or certain dose‐volume parameters on the functional lung).^[^ ^12^, ^20^ ^]^ However, there are some inconsistencies regarding dose‐volume parameters on the total lung. For example, Bates et al. ^[^ ^23^ ^]^ show significantly reduced V_20_ consistent with this study, while Yaremko et al. ^[^ ^11^ ^]^ reported increased total lung V_20_.

In addition, although it is generally shown by these studies that Voxel and FuncAvoid treatment of the lung could be achieved without significantly increasing doses to OARs, such as heart and esophagus, an inconsistency in the PTV dosimetric was observed. For example, Yamamoto et al. ^[^ ^12^ ^]^ report significantly degraded PTV uniformity consistent with the present study, while McGuire et al. ^[^ ^21^ ^]^ show comparable uniformity.

Esophagus dose was set in between 30 and 34 Gy, and it was 40 to 45 Gy for Cord in our optimization model. In Table 2, we see that Voxel‐based plans have slightly higher cord Dmax and esophagus Dmean (p<0.001). This means that some doses were shifted to the critical structures like the cord and esophagus. However, overall, the doses were still well below the dose constraint settings for OARs. The results of this research showed that this type of advanced treatment planning approach should be utilized in clinical settings. In clinical treatment planning, due to the size of the non‐target non‐OAR normal tissue, constraints were not imposed on these normal tissues. In this research, we added another normal tissue constraint to limit the dose shifting from lung region to the critical organs and other non‐target non‐OAR normal tissues. Because we are using an exact mathematical formulation, our approach guarantees the optimal solution under the current formulation. Without being able to directly handle DFH based objective and constraints, due to the limitations of the current treatment planning system, especially the solution approach behind it, we noticed sometimes the anatomical plans generated were not pareto optimal. In addition to the advantage of volume functional areas, we noticed that the overall treatment quality plan can also be improved when voxel‐based optimization techniques are used. That is the additional advantage of our approach.

We now focus on the comparison of our results with those reported in St‐Hilaire et al. ^[^ ^13^ ^]^ due to similar optimization approaches. They conclude functional planning to be more effective in reducing dose to the low‐dose regions of the lung. However, our results show that Voxel treatment planning outperforms both Anatom and FuncAvoid plans in the case of total lung and functional lung. Table 4 shows the reduction of volumes from Anatom plan to FuncAvoid and Voxel plan and FuncAvoid to Voxel plan. The relative improvements in V_*i*_ and fV_*i*_ parameters increase for i=20,..,60. Even though improvement decreases after i=40, the improvements in FuncAvoid over Anatom plan for V_20_ and fV_20_ are insignificant, which is shown in Table 4. Voxel plans reduce volume significantly in all cases compared with both Anatom and FuncAvoid plans.

From our experience we see that voxel‐based plans usually use 2%–4% more MUs than anatomic plans. This is due to the advanced optimization techniques used so that the plan requires slightly more MUs to deliver a higher quality plan. We are not conducting leaf sequencing in this research; therefore, the number of segmentations is not relevant in this research.

## 5 CONCLUSIONS

This research uses a column‐generation approach to generate treatment plans for lung cancer patients. Three treatment plans are generated using real patient data. Results show that Voxel treatment plan improves volume reduction compared with FuncAvoid and Anatom treatment plans.

## CONFLICT OF INTEREST

The authors have declared no conflict of interest.

## Data Availability

The data that support the findings of this study are available on request from the corresponding author. The data are not publicly available due to privacy or ethical restrictions.
